# Serum pharmacokinetics and coagulation aberration induced by sodium dehydroacetate in male and female Wistar rats

**DOI:** 10.1038/srep46210

**Published:** 2017-04-07

**Authors:** Yumei Zhang, Donglai Ying, Hao Liu, Zengrong Yu, Lingling Han, Jiayu Xie, Yang Xie

**Affiliations:** 1 College of Veterinary Medicine, Yangzhou Universitys, Yangzhou, Jiangsu 225009, PR China; 2 Jiangsu Co-innovation Center for Prevention and Control of Important Animal Infectious Diseases and Zoonoses, Yangzhou, Jiangsu 225009, PR China

## Abstract

Sodium dehydroacetate (Na-DHA) is used as a preservative in food, animal feeds and cosmetics. Severe haemorrhage in organs and prolongation of coagulation factors in Sprague–Dawley rats has been reported upon oral administration of Na-DHA. We investigated alterations in coagulation parameters and serum pharmacokinetics upon Na-DHA administration. Wistar rats were administered Na-DHA (50–200 mg/kg, p.o.). Weight gain, food consumption, prothrombin time (PT), activated partial thromboplastin time (APTT), serum levels of Vitamin k (Vk)1, and serum levels of Na-DHA were measured, and histopathology undertaken. Significant reductions in body weight, food consumption and serum levels of Vk1, as well as prolonged PT and APTT, were observed. Females were significantly different from males in terms of serum Na-DHA concentration. Congestion in hepatic sinusoids, renal tubules and spleen, as well as haemorrhage in lung alveoli, gastric mucosa, intestinal mucosa and cardiac muscle cells, were observed by histopathological analyses. Correlation of serum Na-DHA *via* PT and APTT, as well as serum Vk1 *via* PT and APTT, in females was better than that in males. Female rats are more sensitive than males to Na-DHA. Hence, Na-DHA can induce coagulation aberration in Wistar rats, with higher sensitivity seen in females than males.

Sodium dehydroacetate (Na-DHA) is a preservative used widely in foods/beverages, medicines, animal feeds, and cosmetics because it can protect against the growth of bacteria, fungi and yeast^1^,^2^,^3^. Na-DHA is classified as ‘toxic’ or ‘harmful’ only for products for use around the mouth/lips by the European Union (EU)^4^, and is designated as ‘safe’ for general or specific, limited use in food or cosmetics by the EU and US Food and Drug Administration^4^,^5^,^6^. It is also has limited use in food at ≥0.05% in China^7^.

Na-DHA at high concentrations can induce considerable lipid peroxidation and cell damage in normal and α-linolenic acid-loaded cultured rat hepatocytes^8^. Na-DHA (0.2%) added to feed has been reported to affect antioxidant capacity in piglets^9^. Allergic contact dermatitis caused by Na-DHA^10^,^11^,^12^,^13^ and its cytotoxicity^8^ have been reported. Sakaguchi *et al*.^14^ reported that repeated oral administration of Na-DHA in male Sprague–Dawley (SD) rats induced severe haemorrhage in multiple organs and prolongation of blood coagulation factors (BCFs). The effect on coagulation function induced by Na-DHA and its risk assessment have not been adequate and an acceptable daily intake has not been established. Thus, the safety and risk evaluation of Na-DHA used as a preservative must be based on additional toxicology investigations because of these or other side effects.

Warfarin is used in thrombotic diseases because it inhibits the Vitamin k (Vk)-dependent synthesis of BCFs^15^. Anticoagulant drugs such as warfarin are the basis of therapy for venous thromboembolism but can also result in haemorrhage and other side effects^16^. Warfarin has the structure of coumarin (4-hydroxy-2-pyrone), and it has been reported that some derivatives of 4-hydroxy-2-pyrone have anticoagulant activity in rats^17^. The main structure of Na-DHA is 2*H*-pyran-2-one, which is similar to the structure of warfarin. Few studies have focused on the effect of Na-DHA on coagulation apart from haemorrhage in male SD rats^14^.

We investigated the effect of repeated administration of Na-DHA to Wistar rats on changes in body weight, food consumption, parameters of blood coagulation, prothrombin time (PT) and activated partial thromboplastin time (APTT). We also undertook histopathological analyses and studied the serum pharmacokinetics of Na-DHA.

## Results

### Body weight and food consumption

No rats were found to be moribund and no rats died during the study. Changes in body weight and food consumption of rats after administration of different doses of Na-DHA are shown in Table 1 and Table 2. Body weight in females was affected more by Na-DHA administration than in males. In females, body weight decreased significantly 3 days after administration in those receiving 150 or 200 mg/kg Na-DHA (*p* < 0.05), and decreased significantly 7 days after administration in those receiving 50 or 100 mg/kg Na-DHA (*p* < 0.05), compared to that of control. The female body weight increments in 150 and 200 mg/kg groups were all negative during whole test time, that negative weight gain was appeared after 10 days administration in 100 mg/kg females, but negative weight gain was not seen in 50 mg/kg females. In males, body weight in the 200 mg/kg group was significantly lower than that of the control group 3 days after administration (*p* < 0.05), and that in 100 and 150 mg/kg groups decreased significantly 10 days after administration (*p* < 0.05) whereas, in the 50 mg/kg group, a significant difference was not observed (*p* > 0.05). In females, total weight gain in the 50–200 mg/kg group was much lower than that of the control group (especially negative in 150 and 200 mg/kg groups). In males, total weight gain in the 100–200 mg/kg group was significantly lower than that in the control group (*p* < 0.05) (Table 1).

Significant reductions in food consumption were observed since 7 days after administration in females. At 7 d administration, there were significant difference in 50–200 mg/kg groups feed compared to control females (*p* < 0.05), but the food uptake in 200 mg/kg was significantly lower than those in 50, 100 and 150 mg/kg, which was no clear difference in 50–150 mg/kg groups. At 13d, food consumptions in females of tested groups of 50–200 mg/kg groups were all significantly lower than that of control (*p* < 0.05), there were no difference between the four groups. Food consumption in males treated with 50, 100, 150 or 200 mg/kg Na-DHA was decreased significantly 7 days after administration compared with the control group (*p* < 0.05).

### PT and APTT

**Figure 1:**
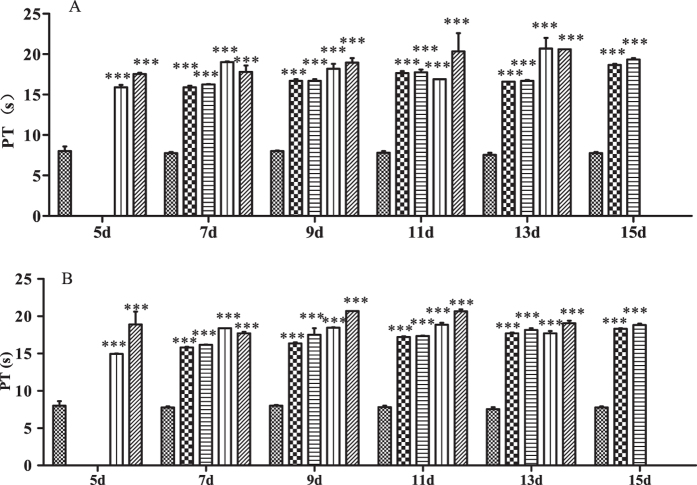
Effect on PT in rats treated with Na-DHA. Wistar rats were administered Na-DHA at different doses for 13 days by lavage. PT values were measured at different times by an automated blood coagulation analyser. (**A**) Male; (**B**) female. 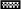 Control, 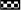 50 mg/kg; 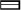 100 mg/kg; 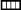 150 mg/kg; 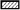 200 mg/kg. ***p < 0.001, significantly different from controls.

**Figure 2:**
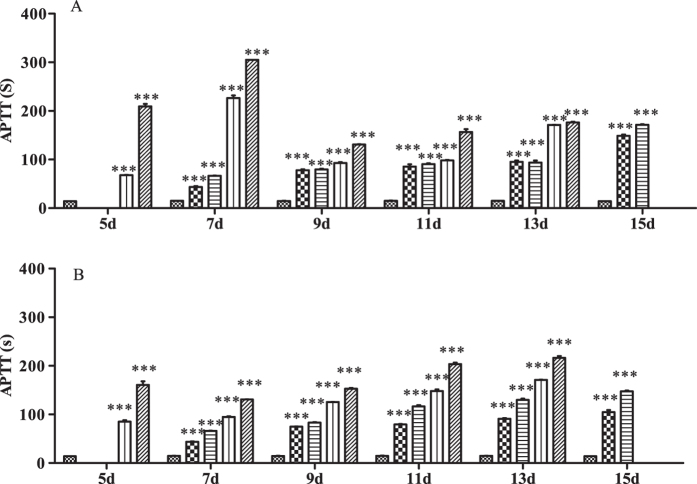
Effect on APTT in rats treated with Na-DHA. Wistar rats were administered Na-DHA at different doses for 13 days by lavage. APTT values were measured at different times by an automated blood coagulation analyser. (**A**) Male; (**B**) female. 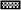 Control, 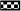 50 mg/kg; 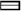 100 mg/kg; 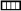 150 mg/kg; 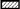 200 mg/kg. ***p < 0.0 01, significantly different from controls.

Data for PT and APTT in rats treated with different doses of Na-DHA are shown in Figs 1 and 2. PT and APTT were prolonged significantly in each rat of 50, 100, 150 and 200 mg/kg groups at each time point post-administration (p < 0.001). There was no clear relationship in dose *via* PT or time *via* PT, and there was no clear difference in PT, between males and females.

APTT in males receiving 200 mg/kg on day 5–7 was 200–300 s, which was longer than that for females in the same dose group (160–130 s). APTT decreased and was maintained at 150–170 s at day 9–15. APTT in females was longer with increasing dose at a single time point or longer with increasing time in a particular dose group within day 7–13. APTT in females administered Na-DHA appeared to have a clear dose–effect and time–effect relationships.

### Serum concentration of Na-DHA

**Figure 3:**
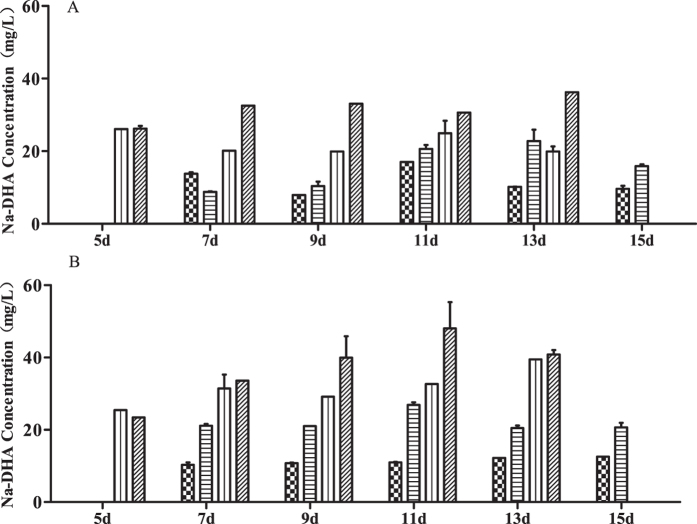
Serum concentration of Na-DHA in rats by HPLC. Serum concentrations of Na-DHA were Analyzed by method after Wistar rats were administered Na-DHA (50–200 mg/kg). (**A**) Male; (**B**) female. 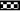 50 mg/kg; 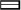 100 mg/kg; 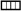 150 mg/kg; 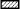 200 mg/kg.

Data for serum levels of Na-DHA are shown in Fig. 3. At 7–13 days, the Na-DHA concentration (in mg/L) in males was 26–36 in the 200 mg/kg group and 20–25 in the 150 mg/kg group whereas, in females, the respective values were 33–48 and 31–40. There were significant differences in serum levels of Na-DHA between males and females administered 100–200 mg/kg except for the 50 mg/kg group (p = 0.1769), with p = 0.0141 in the 200 mg/kg group and p < 0.001 in the 150 and 100 mg/kg groups (ANOVA).

### Serum levels of Vk1

**Figure 4:**
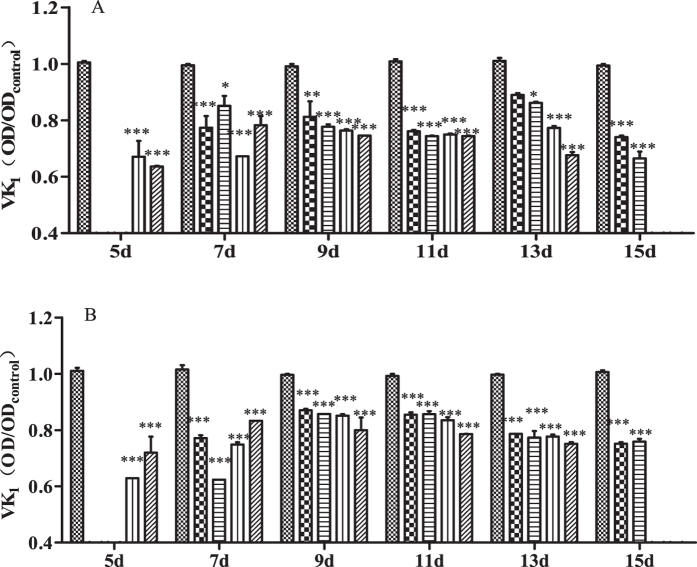
Serum level of Vk1 in rats administered Na-DHA. Serum Vk1 content of rats administered different doses of Na-DHA was measured by ELISA. (**A**) Male; (**B**) female. 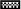 Control, 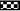 50 mg/kg; 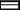 100 mg/kg; 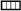 150 mg/kg; 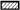 200 mg/kg. *p < 0.05, **p < 0.01, ***p < 0.001, significantly different from controls.

The standard curve for Vk1 was y = 0.8853x + 0.0149 at a concentration of 0.75–6.00 ng/L, where y was OD_450nm_, and x was the Vk1 concentration (ng/L); the coefficient of determination (R^2^) was found to be 0.99898. Serum levels of Vk1 of rats administered Na-DHA are shown in Fig. 4. Serum levels of Vk1 in males and females were decreased significantly at different time points after Na-DHA administration compared with the control group, p value < 0.05–0.001 in males and p < 0.001 in females. There was no significant difference in serum levels of Vk1 between males and females (p = 0.2621).

### Correlation analyses

**Figure 5:**
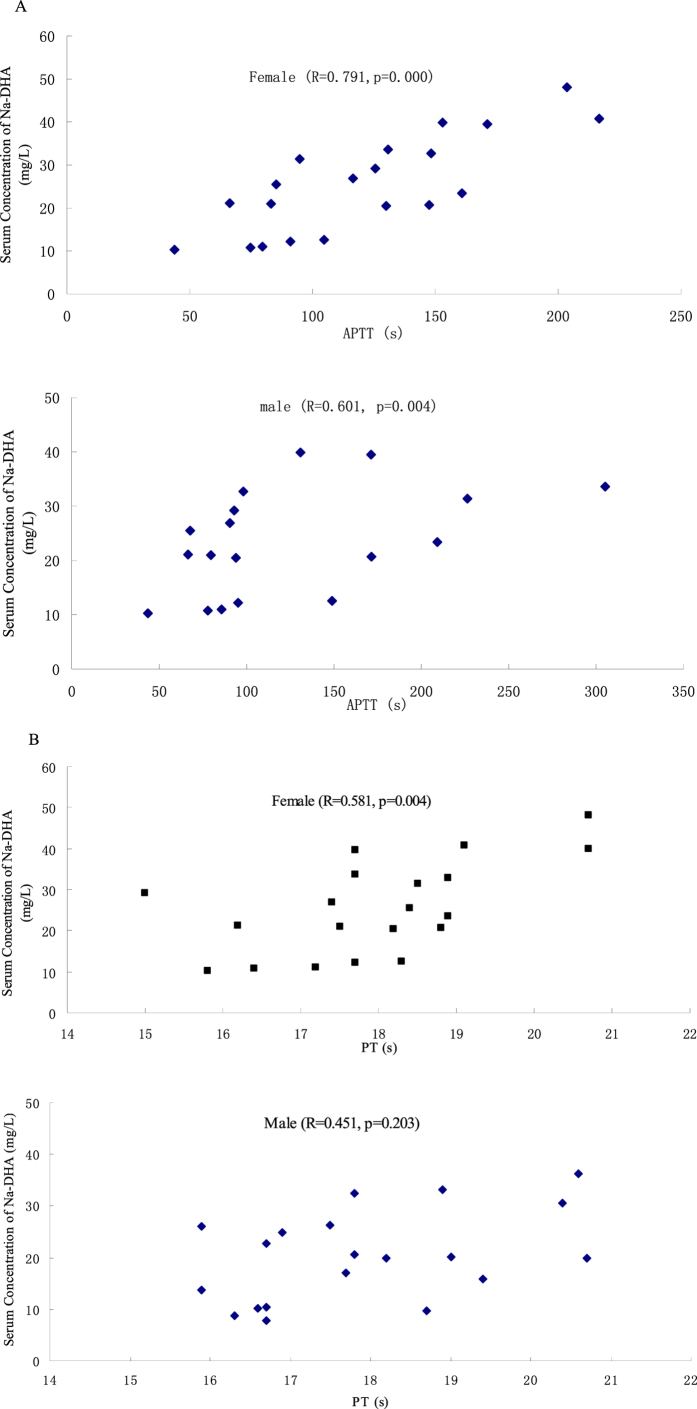
The correlation analysis of serum Na-DHA concentration of rats and the PT or APTT values. Wistar rats were administered Na-DHA at 50–200 mg/kg doses for 13 days by lavage. The bloo dwas taken at 5, 7, 9, 11 and 13 days after the first administration each time point with four males and four females. PT and APTT values were measured at different times by an automated blood coagulation analyser. Serum Na-DHA concentrations at above time points were detected by the HPLC method. The mean values of PT, APTT and Na-DHA serum concentration at each time points were used in Pearson correlation analyses (**A**, Na-DHA *vis* PT; **B**, Na-DHA *vis* APTT).

Correlation between serum levels of Na-DHA and PT as well as APTT was analyzed in Fig. 5. The mean values of PT, APTT and Na-DHA serum concentration four times repetitive at each time points were used in Pearson correlation analyses. The correlation coefficient (R^2^) between serum levels of Na-DHA and PT and APTT in females was 0.581 (p = 0.004) and 0.791 (p = 0.000), respectively. The R^2^ of serum levels of Na-DHA *via* PT was 0.451 (p = 0.203) in males. The R^2^ of the serum levels of Na-DHA *via* APTT was 0.601 (p = 0.004) in males.

The R^2^ between serum levels of Vk1 and PT in females and males was −0.486 (p = 0.030) and −0.188 (p = 0.420), respectively. The R^2^ between serum levels of Vk1 and APTT was −0.203 (p = 0.390) and −0.062 (p = 0.788) in females and males, respectively. There was no correlation between serum levels of Na-DHA and serum levels of Vk1 in females (R^2^ = −0.203, p = 0.390) or males (R^2^ = −0.035, p = 0.884).

### Congestion or haemorrhage in tissues by histopathological analyses

**Figure 6:**
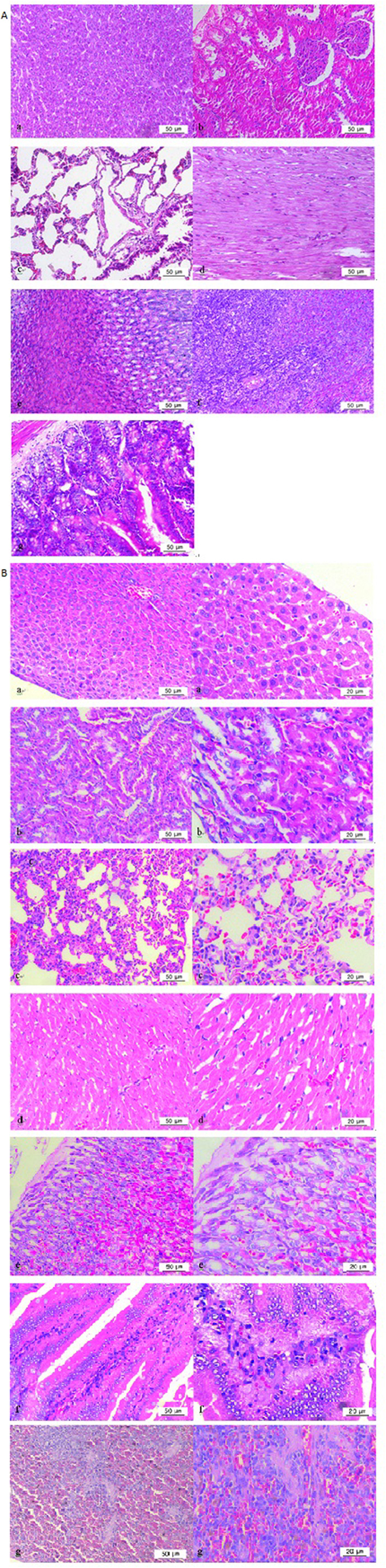
Histopathology of paraffin sections by H&E staining. Tissues were collected 15 days after the first administration of 200 mg/kg Na-DHA, after blood had been taken from the abdominal aorta of rats anesthetised using 2% pentobarbital sodium. (**A**) Normal control (200×); (**B**) (200×) and (400×), 200 mg/kg Na-DHA group; a, liver; b, kidney; c, lung; d, heart; e, stomach; f, intestine; g, spleen.

Occasionally, haemorrhage could be observed by the naked eye in subcutaneous tissue and the liver surface. H&E staining of the tissues of rats treated with 200 mg/kg Na-DHA are shown in Fig. 6, congestion or haemorrhage in tissues was observed. H&E staining also revealed: congestion in hepatic sinusoids, renal tubules and spleen; slight haemorrhage in lung alveoli and intestinal mucosa; considerable haemorrhage in the gastric mucosa and cardiac muscle cells. There was not clear difference between males and females with respect to congestion or haemorrhage of these tissues. Severe haemorrhage was not observed in these organs. Similar congestion or haemorrhage of these tissues was observed upon administration of 50–150 mg/kg of Na-DHA.

## Discussion

Na-DHA (50–200 mg/kg, p.o.) administration caused the body weight and food consumption to decrease, and PT and APTT to prolong significantly, in Wistar rats. These results are similar to those observed when Na-DHA has been administered to male SD rats^14^. According to our data on weight gain, food consumption and APTT, female rats were more sensitive than male rats to Na-DHA administration. Serum levels of Na-DHA in females were significantly higher than those in males when 100–200 mg/kg was administered. Serum levels of Na-DHA in males or females were related more closely with APTT (R^2^ = 0.705 and 0.791, respectively) than with PT in males or females (R^2^ = 0.283 and 0.491, respectively). The correlation R^2^ values of Na-DHA in serum *via* PT and APTT in females (0.491 and 0.791, respectively) were obviously greater than those in males (0.283 and 0.705 respectively). The higher serum concentration in females was in accordance with greater changes in weight gain and food consumption, and prolonged APTT. These results could be explained by higher sensitivity in females than in males based on the relationship between blood concentrations of drugs with pharmacological actions.

The correlation between APTT and Na-DHA serum concentration was significant, PT and serum concentration was not significant in male rats. PT is the prothrombin time which suggests the level of prothrombin innate or acquired, fibrinogen and clotting factors. APTT is the activated partial thromboplastin time, which relates with the factors in intrinsic contact activated pathway, prolong APTT reflects deficiency of contact factors, or factors VIII, IX and XI, or heparin and direct thrombin inhibitors (PT may also prolonged), or disease like lupus anticoagulant^18^. If the contact activated pathway was affected by a chemical compound which was not a direct thrombin inhibitor, the correlation of APTT via dose or serum concentration is more than the correlation of PT via concentration. The lower correlation between PT and Na-DHA serum concentration in male rats whereas better correlation between APTT and Na-DHA serum concentration, implying Na-DHA affect the intrinsic contact activated pathway in Wistar rats and then deficiency of contact factors or some activated factors. The effect of Na-DHA on activated pathway in male may be less than that in female, which the correlation between PT and serum concentration is significant in female rats.

Correlation of Vk1 concentration *via* PT was −0.486 in females and −0.188 in males. Correlation of Vk1 concentration *via* APTT was −0.203 in females and −0.006 in males. These data suggested a stronger correlation of Vk1 *via* PT than Vk1 *via* APTT. Na-DHA administration caused a significant decrease in serum levels of Vk1. Sakaguchi *et al*.^14^ reported Na-DHA-induced haemorrhage to result from Vk deficiency *via* inhibition of vitamin k1 epoxide reductase (VKOR) activity. Oral administration of anticoagulants leads to the synthesis of the Vk-dependent clotting factors II, VII, IX and X, and gamma-glutamyl carboxylase, which is functionally inactive^19^. PT is related mainly to the clotting factors II, VII, X and V, whereas APTT is related mainly to clotting factors X, VIII, IX and VI. This difference could explain (at least in part) the greater correlation of Vk1 *via* PT than Vk1 *via* APTT in rats administered Na-DHA. In addition, Vk2 is known to be a menaquinone (MK). MKs are synthesised mainly by a small number of bacteria, and are characterised by various subtypes (with MK-4 being the most common Vk2 subtype in animals). UbiA prenyltransferase domain-containing protein 1 (UBIAD1) is a human MK-4 biosynthetic enzyme that is not suppressed by warfarin^20^. Mutations in VKORC1 can cause warfarin resistance^21^. When VKOR inhibited by an anticoagulant causes Vk1 deficiency or warfarin-like resistance, a compensatory increase in Vk2 synthesis maintains Vk function, which affects the correlation of Vk1 with clotting parameters such as PT and APTT.

We did not find a correlation between Na-DHA dose and serum levels of Vk1. Coumarin derivatives with a 4*H*-pyran-2-one structure (e.g., warfarin) have been reported to block VKOR activity, cause VK deficiency, and to inhibit coagulation^22^,^23^. Serum levels of Vk1 are related mainly to Vk1 content in the diet, Vk cycling in the liver, and Vk synthesis in intestinal bacteria. The change in Vk1 content induced by Na-DHA inhibition on VKOR activity contributed only slightly to the total Vk1 concentration. Thus, the correlation between Na-DHA dose and serum levels of Vk1 may be a reasonable explanation. How Na-DHA affects the activity/expression of VKOR, VKORC1^24^,^25^ and protein induced by Vitamin k absence/antagonist II (PIVKA II)^26^ in females and males will be studied carefully in our next work.

Sakaguchi *et al*.^14^ compared the anticoagulation effects of Na-DHA in male SD rats only. In their study, 2 rats in the 200 mg/kg group died, whereas no rats in our study died. In the present study, haemorrhage could be observed by the naked eye in subcutaneous tissue and the liver surface, and congestion/slight haemorrhage was documented by histopathological observation. In the study by Sakaguchi *et al*.^14^, severe haemorrhage was observed in the stomach, intestines, testes, and subcutaneous tissue of male SD rats. Hence, the haemorrhage induced by Na-DHA in SD rats was more severe that observed in Wistar rats.

## Conclusions

We found that 50–200 mg/kg Na-DHA can induce a significant reduction in body weight and food consumption, and clear congestion or haemorrhage in various organs. Correlation of serum levels of Na-DHA *via* PT and APTT, as well as serum levels of Vk1 *via* PT and APTT, in females was better than that in males. Female Wistar rats appear to be more sensitive than male Wistar rats to Na-DHA.

## Methods

### Ethical approval of the study protocol

All protocols were reviewed by the Committee for the Ethics of Animal Experiments of Yangzhou University (Yangzhou, China). Experiments were carried out in accordance with the *Regulations for the Administration of Affairs Concerning Experimental Animals in China* and the EU Directive 2010/63/EU for animal experiments.

### Experimental animals and chemicals

Wistar rats (170–200 g) were obtained from the Comparative Medicine Center of Yangzhou University. They were housed in a room under controlled conditions of 22 °C, relative humidity of 40–60%, and a 12-h light–dark cycle. They had free access to a standard diet and tap water.

Na-DHA (purity >99.0%) was purchased from Sigma–Aldrich (Shanghai, China). A water solution of Na-DHA was prepared for administration to rats.

### Animal studies with Na-DHA administration

Rats were divided into five groups according to the Na-DHA dose (at 5 mL/kg body weight) administered: 0, 50, 100, 150 and 200 mg/kg Na-DHA once a day for 13 days *via* the intragastric route. Body weights were measured on the first day of administration (day 1) and days 3, 7, 10 and 13. Food consumption was measured on days 3, 7 and 13.

Four male and four female rats were anesthetised using 2% pentobarbital sodium. Blood from the abdominal aorta was taken 5, 7, 9, 11, 13 and 15 days after the first administration. Blood was mixed immediately at 1/9 the volume of 3.13% trisodium citrate for blood coagulation. Partial clotted blood was centrifuged at 500 × *g* for 10 min at room temperature. Serum was used for determination of Na-DHA content by high-performance liquid chromatography (HPLC). Tissues from the liver, kidneys, lungs, spleen and heart 15 days after administration were collected. Histopathological analyses with haematoxylin & eosin (H&E) staining was carried out using paraffin sections.

### PT and APTT

Coagulation activity in blood was examined based on PT and APTT using commercial kits (PT assay kit, APTT assay kit; Shanghai Sun Biotech, Shanghai, China) on an automated blood coagulation analyser (Coatron M4; TECO, Nrufahm, Germany) according to manufacturer instructions.

PT was measured in re-calcified plasma in the presence of exogenous thromboplastin supplied in the PT assay kit. APTT was measured in re-calcified plasma in the presence of synthetic phospholipids and ellagic acid supplied in the APTT assay kit.

### Serum levels of Na-DHA by HPLC

Serum (0.5 mL) was mixed with 40 μL acetic acid and 3 mL acetonitrile. This mixture was vortex-mixed for 3 min and agitated for 10 min. After centrifugation (750 × *g* for 10 min at room temperature), the upper liquid layer was collected. Acetonitrile (2 mL) was added to the pellet for the second extraction. The upper layer was evaporated using a rotary evaporator. The residue was dissolved in 1.0 mL of the mobile phase, passed through a filter (0.22 μm), and injected into the HPLC system.

HPLC analyses for Na-DHA was based on the method Zhang *et al*.^27^. The HPLC conditions were briefly as follow: a Waters X brige C_18_ column (5 μm, 4.6 × 250 mm, maintained at 30 °C), a mixture (35:65, v/v) of methanol and 0.02% ammonium acetate (pH 5, adjusted with phosphoric acid) as mobile phase at 1.0 ml/min; 10 μL sample volume injection and detected at 293 nm. A standard curve of Na-DHA was y = 23.772x − 1.779, where y is the Na-DHA chromatographic peak area and x is the Na-DHA concentration in mg/mL, R^2^ = 0.9992 at 0.2–5.0 mg/L. The recovery of Na-DHA was 88.2–93.6% at 0.2–5.0 mg/L spiked in blank serum with lower than 5% intra-day and inter-day variation, the limit of detection was 0.05 mg/L. The retention time of Na-DHA in serum was 8.0 ± 0.1 minter. The Na-DHA content in serum was quantified using the formula:


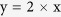


where y is the Na-DHA content (mg/mL) in serum and x is the Na-DHA concentration (mg/mL) based on the standard curve at the final volume.

### Serum levels of Vk1

Serum levels of Vk1 were determined using a rat Vk1 ELISA kit with a limit of detection of 0.1 ng/L. Optical density (OD) was measured at 450 nm using a microplate reader (ELx 800; (BioTek, Shoreline, WA, USA). OD values of test groups were expressed relative to that of the control group.

### Statistical analyses

Parameters were analysed using SPSS v12.0 (IBM, Armonk, NY, USA). Data are the mean ± SD values. One-way analysis of variance (ANOVA) was undertaken to look for significant differences. Pearson analysis was carried out to search for correlations.

## Additional Information

**How to cite this article**: Zhang, Y. *et al*. Serum pharmacokinetics and coagulation aberration induced by sodium dehydroacetate in male and female Wistar rats. *Sci. Rep.* **7**, 46210; doi: 10.1038/srep46210 (2017).

**Publisher's note:** Springer Nature remains neutral with regard to jurisdictional claims in published maps and institutional affiliations.

## Figures and Tables

**Table 1:** Body weights of rats administered Na-DHA.

		Control	50 mg/kg	100 mg/kg	150 mg/kg	200 mg/kg
1 day	♀	174.24 ± 2.185^a^	177.73 ± 2.86^a^	173.96 ± 1.75^a^	169.34 ± 9.76^a^	179.36 ± 12.85^a^
	♂	183.94 ± 2.71^a^	185.51 ± 4.76^a^	182.86 ± 8.72^a^	180.52 ± 1.49^a^	175.79 ± 6.22^a^
3 days	♀	180.67 ± 1.475^b^	178.51 ± 4.01^b^	174.97 ± 1.90^ab^	164.31 ± 3.77^a^	176.36 ± 5.08^b^
	♂	196.44 ± 0.82^a^	199.55 ± 4.76^a^	187.99 ± 8.04^a^	196.35 ± 1.40^a^	181.17 ± 1.15^b^
7 days	♀	191.93 ± 2.605^a^	173.26 ± 1.02^b^	185.07 ± 12.7^a^	163.68 ± 1.00^c^	164.84 ± 1.84^c^
	♂	208.73 ± 0.38^a^	209.45 ± 0.76^a^	183.61 ± 17.91^c^	201.50 ± 4.50^a^	191.78 ± 4.18^b^
10 days	♀	200.07 ± 1.280^a^	182.44 ± 3.18^b^	173.18 ± 2.13^b^	157.10 ± 1.50^d^	171.57 ± 5.24^bc^
	♂	217.40 ± 1.10^a^	229.39 ± 9.71^a^	196.01 ± 8.06^c^	209.41 ± 8.20^b^	191.81 ± 10.67^c^
13 days	♀	216.99 ± 0.465^a^	184.04 ± 4.62^b^	174.76 ± 2.32^c^	154.63 ± 0.42^e^	170.50 ± 3.50^c,d^
	♂	226.86 ± 2.56^a^	237.05 ± 6.74^a^	214.99 ± 8.28^b^	214.00 ± 5.78^b^	196.45 ± 8.31^c^
Total weight gain	♀	42.75 ± 1.68^a^	6.31 ± 3.61^b^	0.80 ± 4.56^b^	−10.71 ± 3.68^c^	−8.86 ± 5.46^c^
	♂	42.92 ± 1.84^a^	41.54 ± 5.26^a^	32.13 ± 8.66^b^	33.48 ± 4.32^b^	20.66 ± 4.84^c^

Different letters on the same line denote significant differences from control, *p < 0.05, whereas the same letters on the same line denote no significant difference (p > 0.05).

**Table 2:** Food consumption of rats administered Na-DHA (g/day/rat).

		Control	50 mg/kg	100 mg/kg	150 mg/kg	200 mg/kg
3 days	♀	18.03 ± 0.61^ab^	19.93 ± 0.06^c^	18.89 ± 0.08^bc^	18.61 ± 0.05^b^	17.26 ± 0.14^a^
	♂	22.99 ± 0.34^abc^	23.81 ± 0.00^c^	22.10 ± 0.05^a^	22.78 ± 0.03^ab^	23.08 ± 0.04^bc^
7 days	♀	21.96 ± 0.41^c^	17.63 ± 0.47^b^	17.32 ± 0.33^b^	17.39 ± 0.22^b^	15.67 ± 0.43^a^
	♂	28.19 ± 0.23^d^	27.78 ± 0.00^c^	25.01 ± 0.01^b^	23.39 ± 0.06^b^	22.22 ± 0.11^a^
13 days	♀	25.35 ± 0.99^b^	7.75 ± 0.09^a^	7.08 ± 0.15^a^	6.11 ± 0.47^a^	6.88 ± 0.75^a^
	♂	34.97 ± 0.67^c^	13.19 ± 0.20^b^	10.81 ± 0.03^a^	9.95 ± 0.02^a^	10.21 ± 0.23^a^

Different letters on the same line denote significant differences from control, *p < 0.05, whereas the same letters on the same line denote no significant difference (p > 0.05).
